# The dehydration- and ABA-inducible germin-like protein CpGLP1 from *Craterostigma plantagineum* has SOD activity and may contribute to cell wall integrity during desiccation

**DOI:** 10.1007/s00425-020-03485-0

**Published:** 2020-10-12

**Authors:** Valentino Giarola, Peilei Chen, Sarah Jane Dulitz, Maurice König, Stefano Manduzio, Dorothea Bartels

**Affiliations:** 1 Institute of Molecular Physiology and Biotechnology of Plants (IMBIO), University of Bonn, Kirschallee 1, 53115 Bonn, Germany; 2 Present Address: Department of Genomics and Biology of Fruit Crops, Research and Innovation Centre, Fondazione Edmund Mach, San Michele all’Adige, Italy; 3 Present Address: College of Life Sciences, Henan Normal University, Xinxiang, 453007 China; 4 Present Address: IZMB, University of Bonn, Kirschallee 1, 53115 Bonn, Germany; 5 Present Address: Institute of Botany, University of Cologne, Zülpicher Straße 47a, 50674 Cologne, Germany; 6 Present Address: Department of Applied Biology, Chonnam National University, Buk-gu, Gwangju, South Korea

**Keywords:** Cell wall remodeling, Desiccation tolerance, Resurrection plants, ROS

## Abstract

**Main conclusion:**

CpGLP1 belongs to the large group of germin-like proteins and comprises a cell wall-localized protein which has superoxide dismutase activity and may contribute towards ROS metabolism and cell wall folding during desiccation.

**Abstract:**

The plant cell wall is a dynamic matrix and its plasticity is essential for cell growth and processing of environmental signals to cope with stresses. A few so-called resurrection plants like *Craterostigma plantagineum* survive desiccation by implementing protection mechanisms. In *C. plantagineum*, the cell wall shrinks and folds upon desiccation to avoid mechanical and oxidative damage which contributes to cell integrity. Despite the high toxic potential, ROS are important molecules for cell wall remodeling processes as they participate in enzymatic reactions and act as signaling molecules. Here we analyzed the *C. plantagineum* germin-like protein 1 (CpGLP1) to understand its contribution to cell wall folding and desiccation tolerance. The analysis of the CpGLP1 sequence showed that this protein does not fit into the current GLP classification and forms a new group within the Linderniaceae. *CpGLP1* transcripts accumulate in leaves in response to dehydration and ABA, and mannitol treatments transiently induce *CpGLP1* transcript accumulation supporting the participation of CpGLP1 in desiccation-related processes. CpGLP1 protein from cell wall protein extracts followed transcript accumulation and protein preparations from bacteria overexpressing CpGLP1 showed SOD activity. In agreement with cell wall localization, CpGLP1 interacts with pectins which have not been reported for GLP proteins. Our data support a role for CpGLP1 in the ROS metabolism related to the control of cell wall plasticity during desiccation in *C*. *plantagineum*.

**Electronic supplementary material:**

The online version of this article (10.1007/s00425-020-03485-0) contains supplementary material, which is available to authorized users.

## Introduction

Plants are often subjected to rapid environmental changes during their life cycle. The plant cell wall, a complex matrix of polysaccharides and proteins, provides shape, support, and protection to cells. Cell walls are not static structures, and they must be flexible to permit cell growth, but they must also form barriers to protect cells from pathogen infections and mechanical damage. Cell wall plasticity is the capacity of the cell wall to dynamically respond to developmental and environmental cues (Chen et al. 2019). The composition of the wall polysaccharide chains, their proportions and their interconnections strongly influence cell wall plasticity. Specific cell wall proteins contribute to the functions of cell walls required in different tissues, developmental stages or environmental interactions. The activation or recruiting of cell wall remodeling systems including cell wall-modifying enzymes and Ca^2+^ ions permit plants to change the rheological properties of their cell walls. Control of ROS metabolism at the cell wall is essential for remodeling mechanisms as ROS such as H_2_O_2_ participates in the enzymatic activity of cell wall-modifying enzymes and may also act as signaling molecule in plant–pathogen interactions at the cell wall (Schmidt et al. 2016).

Resurrection plants are a group of several hundred angiosperm plants which can tolerate desiccation in their vegetative tissues (Gaff and Oliver 2013). In resurrection plants, cell walls extensively shrink and fold upon desiccation, but the integrity and continuity of wall structures are maintained and restored when tissues are re-watered (Farrant 2000; Farrant et al. 2003; Willigen et al. 2003). The capacity to survive extreme folding, i.e., to prevent damage due to mechanical stress generated between the protoplasm and the cell wall, must have been acquired through polysaccharide/protein cell wall complexes and the induction of cell wall complex formations. Dehydration-induced changes in the cell wall architecture and composition as well as the presence of pectin-associated arabinans have been linked to cell wall plasticity in resurrection species (Moore et al. 2006, 2008; Vicré et al. 1999, 2004). Our understanding of cell wall folding mechanisms in resurrection plants is puzzling as species seem to have evolved species-specific strategies to cope with reversible shrinking and folding. Some species like *Myrothamnus flabellifolia* rely on high constitutive levels of pectic-arabinans, arabinogalactan–proteins and arabinoxylans, whereas others, such as *Craterostigma* spp., adjust cell wall plasticity via dehydration-induced biochemical reactions (Jung et al. 2019; Moore et al. 2008, 2013). Among such mechanisms, the reduction of the hemicellulose xyloglucan content and modifications of pectin structures have been described (Jung et al. 2019; Vicré et al. 1999, 2004). Monoclonal antibodies directed against pectin and hemicellulose epitopes demonstrated dehydration-induced changes in rhamnogalacturonan I, rhamnogalacturonan II and hemicelluloses and a reduction of pectin methylesterification (Jung et al. 2019).

*CpCRP1*, *CpGRP1*, *CpWAK1*, and *pcC13-62* genes encode cell-wall-localized proteins in the resurrection plant *C. plantagineum* (Chen et al. 2019; Giarola et al. 2015b). The abundance of the proteins is modulated during the dehydration and rehydration cycle and thus these proteins are candidates to participate in cell wall folding mechanisms (Giarola et al. 2015b, 2016, 2018; Jung et al. 2019). A yeast two-hybrid screening identified germin-like proteins among cell wall interacting proteins in *C. plantagineum* (Dulitz 2016).

Germins and Germin-like proteins (GLPs) belong to the cupin superfamily which includes ubiquitous and biochemically diverse proteins containing a six-stranded beta-barrel structure referred to as cupin domain (Khuri et al. 2001). Plant germins and GLPs are phylogenetically separated from other cupins and there is a correlation between protein conservation and function (Khuri et al. 2001). Some GLPs are bi-functional enzymes with both oxalate oxidase (OXO) and manganese superoxide dismutase (Mn-SOD) activities (Woo et al. 2000), others showed only Mn-SOD activity (Pei et al. 2019). Bi-functional germins are often named “true germins” and cluster together in phylogenetic trees (Carter and Thornburg 1999). GLPs have been described to be circadian or developmentally regulated or expressed upon biotic and abiotic stresses (Davidson et al. 2009; Dunwell et al. 2008). For example, the cell wall-associated *Sinapis alba* L. GLP is differentially expressed during the day/night cycle (Heintzen et al. 1994) whereas the expression of the pine GLP PcGER1 is linked to embryogenesis (Domon et al. 1995; Neutelings et al. 1998). The *Nectarin I* gene is almost exclusively expressed in tobacco nectar where it is the most abundant protein (Carter and Thornburg 2000). Germin-like transcripts accumulate upon powdery mildew infections in wheat or fire blight apple infections whereas in barley HvGER4d accumulates exclusively upon *Blumeria graminis* infection (Bonasera and Beer 2002; Schweizer et al. 1999; Zimmermann et al. 2006). Among abiotic stresses, salt, mechanical wounding, drought, cold, aluminum and high temperatures were shown to induce, inter alia, the accumulation of GLPs (Davidson et al. 2009; Hamel et al. 1998; Hurkman et al. 1991; Jiang et al. 2007; Lu et al. 2010; Vallelian-Bindschedler et al. 1998). Half of the rice germins are induced by either both biotic and abiotic factors or by pathogen infection only (Davidson et al. 2009). Two of them, Os01g72290 and Os03g44880, are induced by drought stress whereas OsGER5 protein is down-regulated upon drought (Davidson et al. 2009; Ke et al. 2009). Stress-responsive GLPs from different taxa generally cluster together and are predicted to be MnSODs (Carter and Thornburg 2000; Khuri et al. 2001). A possible role of germin-like proteins in dehydration is also supported by the observation that transcripts coding for a germin-like protein similar to the *Arabidopsis thaliana* germin-like protein GLP5A (P92996) were found to accumulate in dried leaves of the resurrection plant *Boea hygrometrica* (Wang et al. 2009). GLPs and germins are built on average of 220 amino acids and contain three conserved amino acid motifs often referred to as germin Boxes A, B, and C (Barman and Banerjee 2015; Bernier and Berna 2001). Boxes B and C are separated by a 15- to 26-amino-acid intermotif region and contain three conserved histidine residues and one conserved glutamate (Dunwell et al. 2000; Woo et al. 2000). Ancestral relatives have intermotif regions shorter than modern relatives such as cereal GLPs (Khuri et al. 2001). Two conserved cysteines at the N terminus form an internal disulphide bridge and delimit a hypervariable amino acid region (Bernier and Berna 2001). Germins form a homohexamer (a trimer of dimers) and the three conserved histidines and a glutamate in the B and C boxes are ligands for a single manganese ion in the active site of each monomer (Requena and Bornemann 1999; Woo et al. 2000). This compact beta-barrel structure permits germins to remain functional in extreme conditions (Thompson and Eisenberg 1999). Amino acid ligands of manganese are shared with MnSOD enzymes although the overall protein fold of MnSODs and cupins is different. GLPs are divided into eight GLP subfamilies and two bryophyte subfamilies according to sequence similarity (Barman and Banerjee 2015; Lu et al. 2010; Nakata et al. 2004; Zimmermann et al. 2006). SOD activity has been demonstrated for a limited number of GLP subfamily 1, subfamily 2, subfamily 5/6, subfamily 7 and bryophyte subfamily 1 and 2 members (Barman and Banerjee 2015).

OXO GLPs are active at low pH and are likely to be involved in the detoxification of oxalic acid produced by plant pathogens. The SOD function of GLPs is active at neutral pH and should protect plants from biotic- and abiotic-induced oxidative stress (Dunwell et al. 2000; Khuri et al. 2001). Besides the metal-binding domain, an N-glycosylation and an N-terminal signal peptide for cell wall localization are usually present in GLPs (Bernier and Berna 2001).

The objective of this study was to investigate the *C. plantagineum CpGLP1* gene at the transcript and protein level to provide a clue how GLP1 may contribute to cell wall properties required for desiccation tolerance in *C. plantagineum*. *CpGLP1* transcripts accumulate in response to dehydration and are transiently induced upon ABA and mannitol treatments in leaves. The CpGLP1 protein accumulates in protein extracts enriched for cell wall proteins thus supporting an extracellular localization. CpGLP1 weakly interacts with pectins and has SOD activity suggesting that it may be involved in oxidative stress management linked to cell wall protection/remodeling during dehydration.

## Materials and methods

### Plant materials and treatments

*C. plantagineum* Hochst. plants were grown according to Bartels et al. (1990). Plants were dried and rehydrated in pots for the indicated time. The relative water content (RWC) of dehydrating leaves was determined according to Bernacchia et al. (1996). For osmotic and ABA treatments, fully grown leaves of well-watered plants were detached and incubated for the indicated time in 0.5 M mannitol or 100 µM ABA, respectively. Leaves, incubated in water, were used as control. Leaf tissues were ground in liquid nitrogen and stored at − 80 °C or freeze-dried.

### Identification of CpGLP1 homologs and phylogenetic analysis

**Fig. 1:**
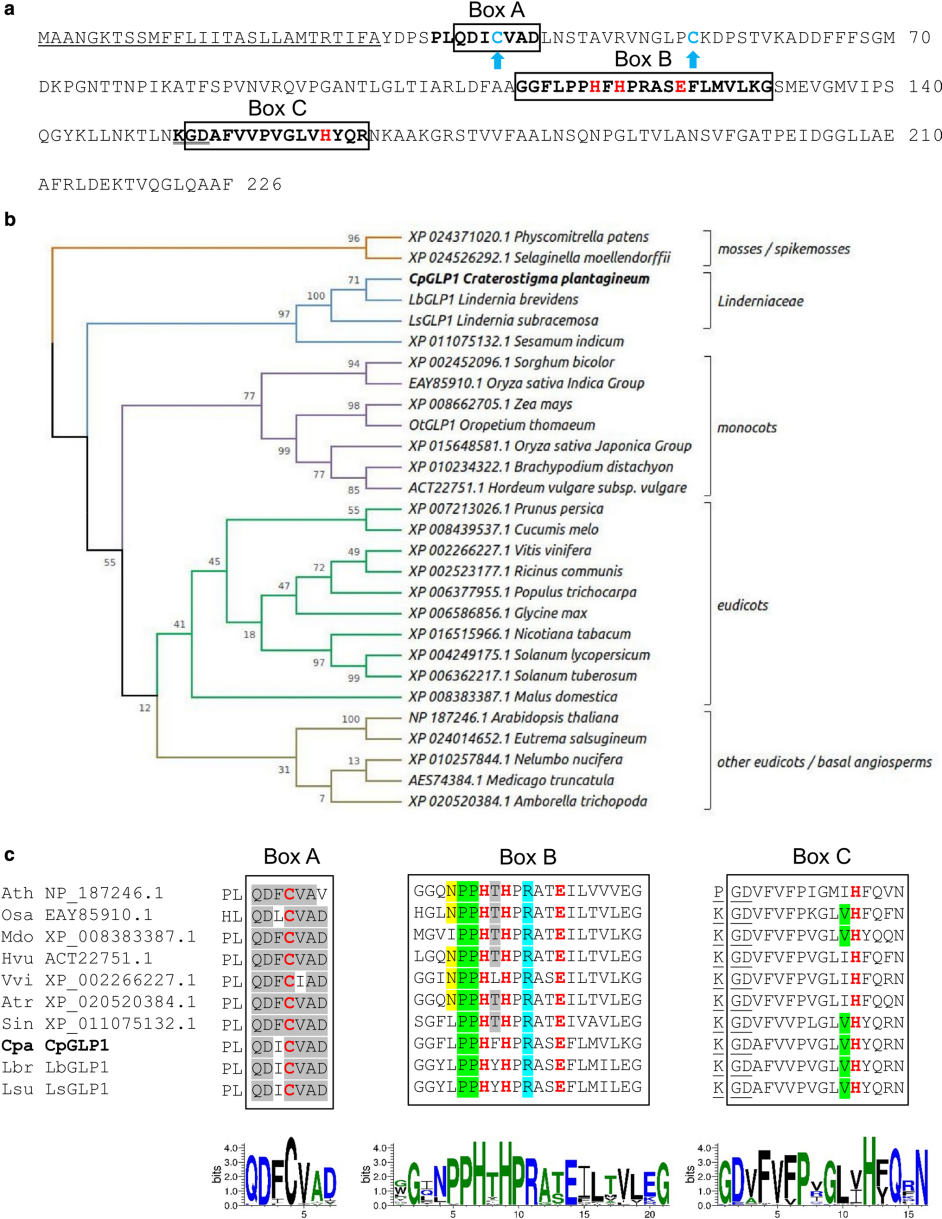
Analysis of the *Craterostigma plantagineum* germin-like protein 1 (CpGLP1) sequence. **a** CpGLP1 predicted protein sequence. The *CpGLP1* sequence was retrieved from our transcriptome databank and cloned to confirm sequence identity. The three sequence motifs which are usually found in germin and germin-like proteins are indicated with rectangular boxes (Boxes A, B, and C) (Barman and Banerjee 2015). The two cysteines that form an internal disulfide bridge are indicated with blue arrows and the four amino acids (H, H, E, H) required for the metal ion binding are shown in red. The predicted signal peptide sequence and the KGD motif are underlined with a single and double line, respectively. **b** Phylogenetic analysis of CpGLP1 homologs inferred using the maximum likelihood method based on the JTT matrix-based model (Jones et al. 1992). The percentage of trees in which the associated taxa clustered together is shown next to the branches. Initial tree(s) for the heuristic search were obtained automatically by applying neighbor-joining and BioNJ algorithms to a matrix of pairwise distances estimated using a JTT model, and then selecting the topology with superior log likelihood value. Evolutionary analyses were conducted in MEGA X (Kumar et al. 2018). **c** Conservation of amino acid sequence in the three boxes typical of germin and germin-like proteins. Selected CpGLP1 homologs were aligned and the sequences corresponding to Box A, Box B and Box C are shown. Amino acids involved in disulfide bonds and metal binding (see a) are indicated in red and amino acids which are conserved in GER 5 and GER 6 classes (Barman and Banerjee 2015) are underlined with colors within the boxes. Sequence logos are shown below sequence boxes to indicate the overall sequence conservation of CpGLP1 homologs. Logos were generated using WebLogo 3 (https://weblogo.threeplusone.com/)

Proteins similar to the *C. plantagineum* germin-like protein 1 (CpGLP1; GenBank accession number MT978083) were identified from *Lindernia brevidens*, *Lindernia subracemosa* and *Oropetium thomaeum* transcriptome data (VanBuren et al. 2015, 2018) or GenBank using the CpGLP1 predicted protein sequence as the query. CpGLP1 homologs were aligned with T-Coffee (https://tcoffee.crg.cat/apps/tcoffee/do:regular) (Notredame et al. 2000) and the sequence alignment was input in MEGA X (Kumar et al. 2018) to reconstruct the evolutionary gene history. The phylogenetic analysis was conducted using the Maximum Likelihood method based on the JTT matrix-based model (Jones et al. 1992). The tree with the highest log likelihood (− 7086.76) is shown in Fig. 1b. The full sequence alignment between *Lindernia* GLPs and selected similar GLPs from GenBank (Fig. S1) was used to generate alignments of germin Boxes A, B, and C which are shown in Fig. 1c. Sequence alignment of CpGLP1 homologs was used to generate sequence logos using WebLogo 3 (https://weblogo.threeplusone.com/) (Fig. 1c). *Lindernia* GLPs were also aligned to GLPs from different groups to generate a phylogenetic tree as previously described in Barman and Banerjee (2015) (Fig. S2).

### Molecular techniques and sequence analysis

Molecular techniques were performed as described in Sambrook et al. (1989). Sequencing of DNA and primer synthesis were carried out by Eurofins MWG Operon (https://www.eurofinsgenomics.eu). Protein localization was predicted using the online tools SignalP v. 5.0 (https://www.cbs.dtu.dk/services/SignalP/) (Almagro Armenteros et al. 2019b) and TargetP-2.0 (https://www.cbs.dtu.dk/services/TargetP/index.php) (Almagro Armenteros et al. 2019a).

### Transcript analysis

**Fig. 2:**
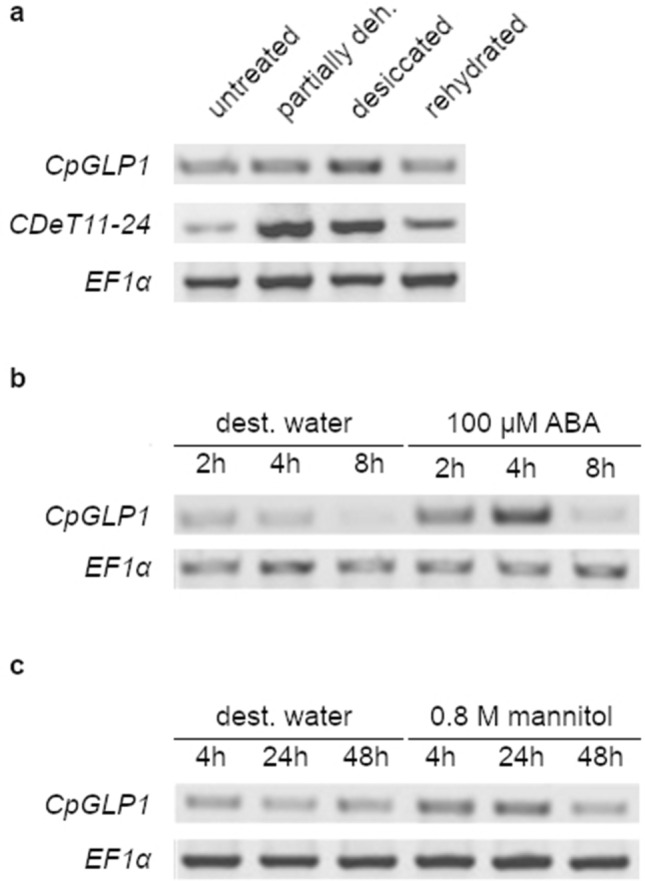
*CpGLP1* transcript expression analysis. **a** Dehydration and rehydration treatments. *C. plantagineum* plants were submitted to a dehydration-rehydration treatment. Samples were taken from untreated (100% relative water content; RWC), partially dehydrated (50% RWC), desiccated (2% RWC) and rehydrated (24 h) leaves. **b** Abscisic acid (ABA) treatment. Fully grown leaves were detached from plants and incubated for 2 h, 4 h and 8 h in a 100 µM ABA solution or in water as control. **c** Osmotic stress treatment. Fully grown leaves were detached from plants and incubated for 4 h, 24 h and 48 h in a 0.8 M mannitol solution or in water as control. Expression levels of the elongation factor 1α (*EF1α*) transcript were analyzed in all samples to monitor differences in input cDNA template. Expression of the dehydration-inducible *CDeT11-24* transcript was measured as a control for the dehydration rehydration treatment (**a**). PCR products were obtained by either 30 (*EF1α* and *CDeT11-24*) or 32 (*CpGLP1*) amplification cycles

Total RNA was extracted according to Valenzuela-Avendaño et al. (2005). Determination of RNA quality and preparation of cDNA were performed as described in Giarola et al. (2015a) with the following modifications. The amount of total RNA used for DNase I treatment was equal to 500 ng and cDNA samples were prepared from 250 ng of DNase I-treated RNA and finally diluted with water to a final volume of 100 µl. PCRs were performed with 5 µl of diluted cDNA samples. The expression level of CpGLP1 in cDNA samples was determined by RT-PCR with CpGLP1_F (5′-TAAACAAGGGCGATGCTTTC-3′) and CpGLP1_R (5′-CAAAGGTGGGCACTAAATGAA-3′) primers. Primers specific for the elongation factor 1α (*CpEF1α*; 5′-AGTCAAGTCCGTCGAAATGC-3′ and 5′-CACTTGGCACCCTTCTTAGC-3′) were used to monitor technical variations in cDNA samples. PCRs were repeated with different settings to identify the best amplification conditions for each gene. PCR products shown in Fig. 2 were obtained by either 30 (*EF1α* and *CDeT11-24*) or 32 (*CpGLP1*) amplification cycles.

### CpGLP1 protein overexpression and production of antiserum

The protein-coding sequence without most of the predicted signal peptide of *CpGLP1* (603 bp, 200 aa) was previously cloned in the yeast two-hybrid vector pAD and was excised from pAD using *Eco*RI and *Sal*I restriction enzymes. The *Eco*RI/*Sal*I CpGLP1 fragment (Fig. S4) was subcloned into the expression vector pET28a( +) (Novagen, Darmstadt, Germany) to obtain the N-terminal 6His-tag translational fusion protein CpGLP1His. The correct pET28-CpGLP1His plasmid was confirmed by DNA sequencing and introduced into BL21 (DE3) *E. coli* cells (Amersham Pharmacia Biotech, Piscataway, NJ, USA) for protein overexpression. BL21 cells carrying the empty pET28 vector were used as a control for expression experiments. *E. coli* cell cultures carrying the pET28-CpGLP1His and the empty pET28 vector (control) were grown to a density of 0.5 at OD_600_ and protein overexpression was induced with 1 mM isopropyl-1-thio-b-d-galactopyranoside (IPTG) for 5 h in the dark at 180 rpm and 26 °C. Pellets from 1 and 100 ml bacterial culture were collected by centrifugation and used for SOD activity assays and affinity chromatography, respectively. For affinity chromatography, inclusion bodies were isolated from bacterial pellets (Schmidt et al. 1986) and dissolved in equilibration buffer in 8 M urea/0.1 M sodium phosphate buffer (pH 8.0). Resuspended proteins were filtered through a 0.2-µm filter (Filtropur S; SARSTEDT, Nümbrecht, Germany) and then purified with the His-Select^®^ Nickel Affinity Gel (Sigma-Aldrich, Saint Luis, MO, USA) under denaturing conditions following the manufacturer’s instructions. CpGLP1His protein was eluted with extraction buffer [0.1 M Hepes (pH 7.9), 6 M urea, 0.5 M imidazole], dialyzed against 6 M urea using a Slide-A-Lyzer™ 10 K Dialysis Cassette (Thermo Fisher Scientific, St Leon-Rot, Germany) and then sent to Seqlab (Sequence Laboratories Göttingen GmbH, Göttingen, DE) to raise a polyclonal antiserum in rabbits. The specificity of the antibody was confirmed using recombinant proteins in protein immunoblots (Fig. S3).

### Protein analysis

Total proteins were extracted from ground leaf material (50–200 mg; according to the leaf RWC) with SDS sample buffer (Laemmli 1970). Cell wall proteins were extracted from 5 g of ground leaf material according to Printz et al. (2015). The CaCl_2_, EGTA and LiCl cell wall extracts were concentrated with Amicon^®^ Ultra 4 ml Centrifugal Filters (Merck KGaA, Darmstadt, Germany), and then precipitated with 4 volumes of cold acetone at − 20 °C. Protein precipitates were dissolved in SDS sample buffer (Laemmli 1970) for protein analysis. For SOD in-gel activity tests, proteins were extracted from bacterial pellets under “semi-native” conditions using a modified SDS sample buffer (without reducing agents) at room temperature (no boiling). Proteins were separated by 10, 12 or 15% (w/v) SDS–poly-acrylamide gel electrophoresis (PAGE) according to Laemmli (1970). SDS gels were either stained with Coomassie blue to monitor equal protein loading or blotted to nitrocellulose membranes for protein immunodetection (Towbin et al. 1979) or used for SOD in-gel activity tests (see below). A 1:5,000 dilution of the polyclonal antiserum was used to detect CpGLP1 protein on nitrocellulose membranes.

### Pectin extraction and estimation of protein concentrations

Pectins were extracted from freeze-dried leaf material (3 mg) with CDTA (1,2-cyclohexanediaminetetraacetic acid) according to Cornuault et al. (2014) with modifications. Briefly, samples were incubated with 1 ml of 50 mM CDTA, pH 7.5, for 1 h on a shaking platform. Supernatants were collected by centrifugation (16,000 *g* for 15 min) and stored at − 20 °C. The galacturonic acid content of the CDTA fractions was determined according to Verma et al. (2014) and used to check for equal extraction. For that, CDTA fractions (400 µl) were mixed with 2.4 ml of 75 mM sodium tetraborate in H_2_SO_4_ and heated in a water bath at 100 °C for 15 min. Samples were cooled in an ice bath for 10 min and mixed thoroughly by vortexing with 80 µl of m-hydroxydiphenyl solution (80 µl of 0.5% NaOH as blank). Absorbance at 525 nm was measured with a spectrophotometer after 5 min incubation at room temperature and the galacturonic acid content was deduced from a standard curve using commercial polygalacturonic acid (Sigma 81325).

### ELISA binding assay

The ELISA binding assay was performed according to Decreux and Messiaen (2005) with modifications. Nunc Maxisorp flat-bottom plate wells (Invitrogen, CA, USA) were coated with commercial pectin from citrus peel (P9135; Sigma-Aldrich, USA; https://www.sigmaaldrich.com) or pectin extracted from *C. plantagineum* leaves (250 µg ml^−1^, 100 µl well^–1^) at 4 °C overnight and then wells were incubated with the following solutions for the indicated time: 100 µl of 3% (w/v) low fat dried milk in wash buffer (20 mM Tris–HCl, 150 mM NaCl, pH 8.0) for 2 h; 50 µl of purified His-tagged CpGLP1 protein in binding buffer (1% low fat dried milk, 20 mM Tris–HCl, 150 mM NaCl, 2 mM CaCl_2_, pH 8.0) for 2 h; wash buffer (four times, briefly); 50 µl of anti-His tag antibody (1:10,000) (Jung et al. 2019) in incubation buffer (1% low fat dried milk, 20 mM Tris–HCl, 150 mM NaCl, pH 8.0) for 1 h; wash buffer (four times, briefly); 50 µl of goat anti-rabbit IgG peroxidase antibody (1:10,000) (Sigma, A9169) in incubation buffer for 1 h; wash buffer (six times, briefly). The bound recombinant CpGLP1 protein was visualized in the presence of the TMB (3,3′,5,5′-tetramethylbenzidine) substrate (Sigma, T2885). The absorbance was measured at 450 nm after color development in the dark and the reaction was stopped by adding 50 µl of 10% (v/v) phosphoric acid.

### SOD activity assay

SOD activity was analyzed in-gel using protein extracts from overexpressing bacteria according to the method described by Beauchamp and Fridovich (1971) and Rucińska et al. (1999).

### Sequence data

*CpGLP1*, *LbGLP1*, *LsGLP1*, and *OtGLP1* sequences were deposited in the NCBI GenBank database under accession numbers MT978083, MT978084, MT978085, and MT978086, respectively.

### Statistical analysis

**Fig. 3:**
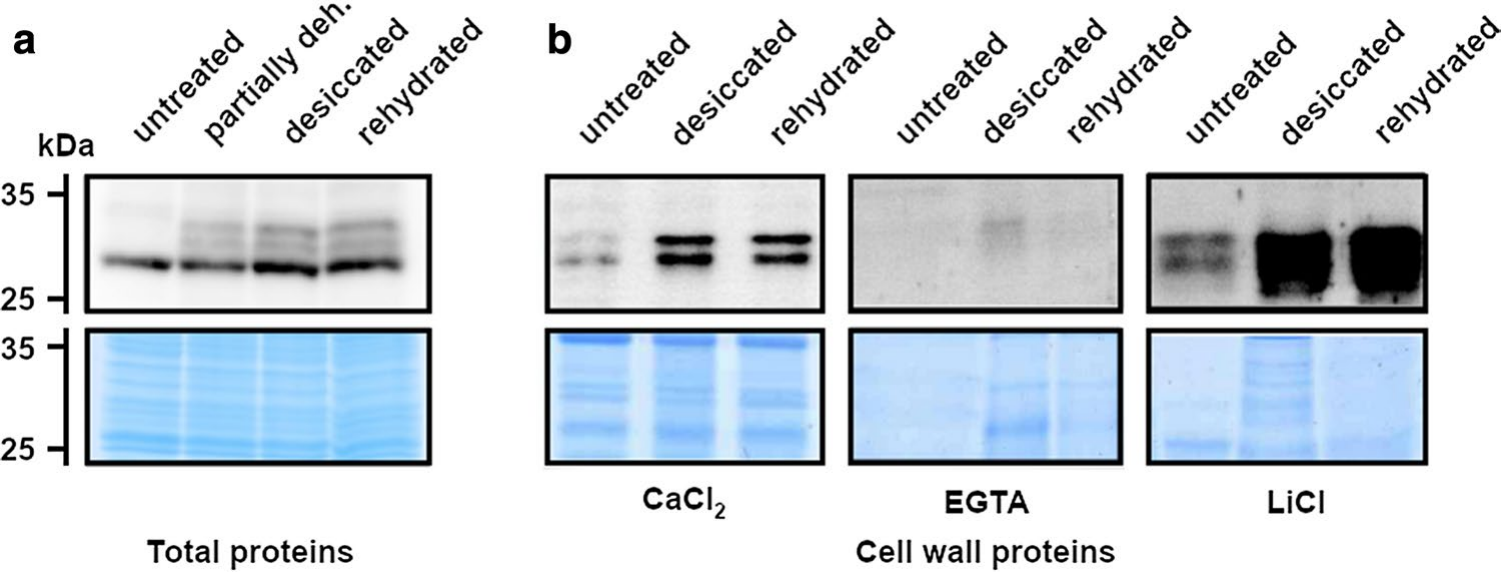
CpGLP1 protein expression. **a** Analysis of CpGLP1 protein accumulation was detected in proteins blots using **a** total proteins and **b** cell wall proteins. Total protein samples were prepared from untreated (100% relative water content; RWC), partially dehydrated (50% RWC), desiccated (2% RWC) and rehydrated (24 h) leaves. Cell wall proteins were prepared from 5 g using the same untreated, desiccated and rehydrated plant material used for total protein preparations. Briefly, plant material was subjected to sequential washes in increasing sucrose concentrations, and then cell wall proteins were extracted with CaCl_2_, EGTA and LiCl according to Printz et al. (2015). CaCl_2_, EGTA and LiCl fractions were concentrated by centrifugation and proteins were precipitated with acetone, resuspended in protein sample buffer and analyzed by 12% SDS-PAGE. Proteins on SDS gels were transferred to a nitrocellulose membrane for immunodetection or stained with Coomassie blue. Immunoblot analysis was performed with 1:5000 dilutions of the CpGLP1 antiserum

**Fig. 4:**
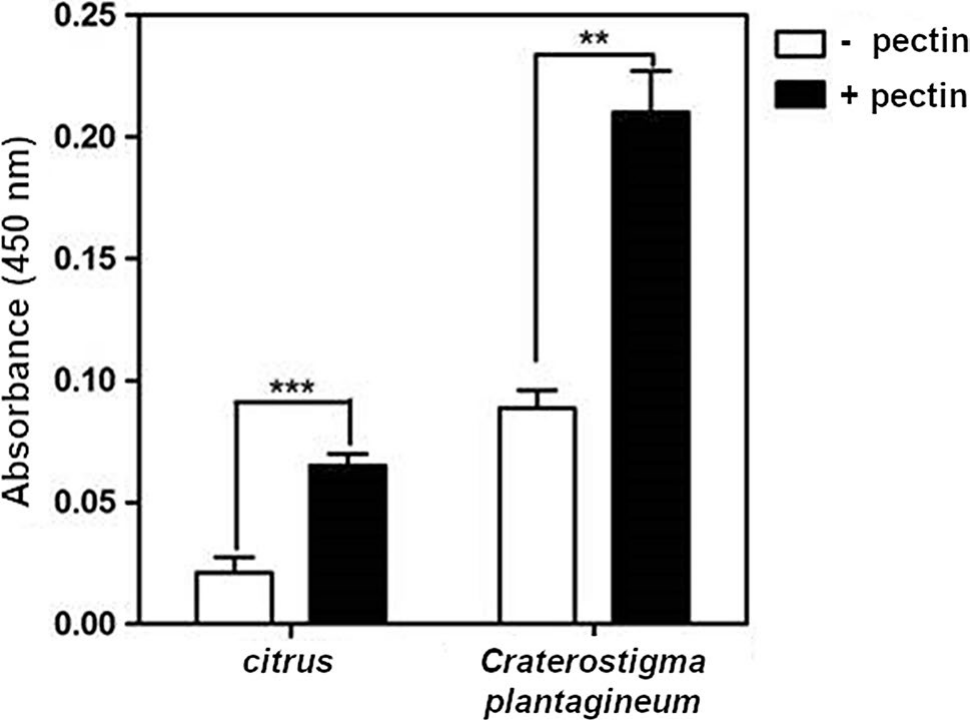
CpGLP1 protein–pectin interaction assays. ELISA plate wells were coated with solutions supplemented with pectin from citrus peel (P9135; Sigma-Aldrich, Saint Luis, MO, USA) and from *C. plantagineum* untreated leaves or without pectin. After coating and blocking the wells, wells were incubated with 0.2 µg of purified recombinant CpGLP1 protein. The bound CpGLP1 proteins were detected with His-tag antibodies. Mean absorbance values were calculated from three technical and three biological repetitions (mean ± SEM). Asterisks above bars denote statistically significant differences compared to control (− pectin) samples (*t*-test, **P < 0.01, ***P < 0.001)

**Fig. 5:**
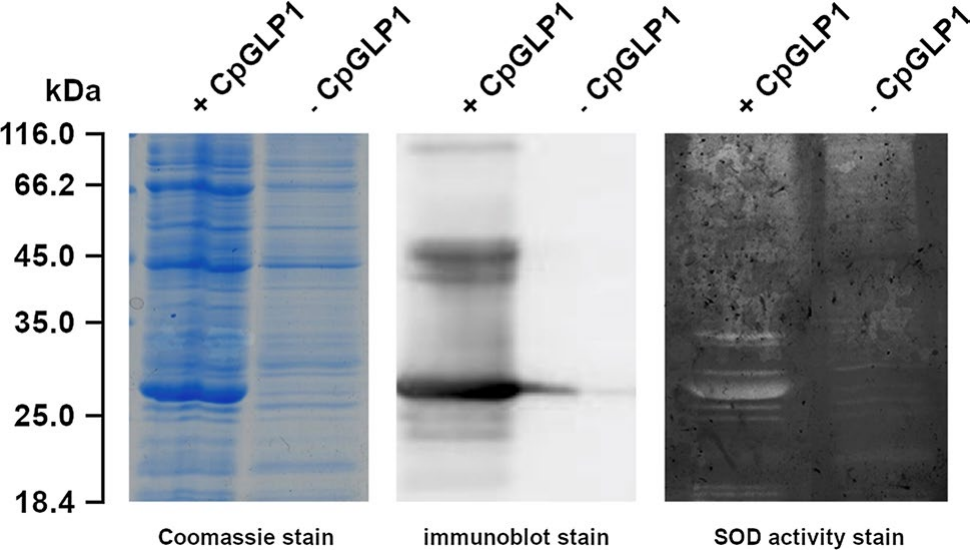
Analysis of SOD activity. Total proteins were extracted from IPTG-induced BL21 cells harboring the CpGLP1 overexpression vector (+ CpGLP1) or the empty vector (− CpGLP1). Protein samples were separated on 12% SDS gels and gels were either stained with Coomassie blue staining total proteins, or blotted on a nitrocellulose membrane for immunodetection, or used for in-gel SOD enzymatic activity test

Transcript and protein expression analyses, and SOD activity tests were done in triplicate (biological replicates) and one representative picture from these analyses is shown in Figs. 2, 3, and 5, respectively. ELISA binding experiments were repeated trice using three biological replicates (Fig. 4). In ELISA binding experiments, mean and standard error of the mean (SEM) values from three biological and three technical replicates (*n* = 9) were calculated and used to determine statistically significant differences between protein–pectin (+ pectin) and control (− pectin) samples by *t* tests. Statistical analyses were performed in GraphPad Prism 5.0 (San Diego, CA; https://www.graphpad.com/).

## Results

### The *C. plantagineum* germin-like protein 1 (*CpGLP1*) gene

Screenings for proteins interacting with dehydration-induced cell wall proteins in *C. plantagineum* identified a germin-like protein (Dulitz 2016). We cloned the full-length cDNA sequence of the corresponding gene and named it *Craterostigma plantagineum* germin-like protein 1 (CpGLP1). *CpGLP1* encodes a protein of about 21 kDa (without the predicted signal peptide) with an isoelectric point of 8.59. The protein sequence of CpGLP1 contains three motifs characteristic for germin and germin-like proteins (Fig. 1a, c). Searches in public databanks identified proteins similar to CpGLP1 in mosses, monocots, and dicots. Putative homologs of *CpGLP1* were identified in transcriptomic data from *Lindernia brevidens* and *Lindernia subracemosa*, two species closely related to *C. plantagineum* but differing in desiccation tolerance (Phillips et al. 2008). The predicted phylogenetic history of CpGLP1 and CpGLP1 homologs is shown in Fig. 1b. The GLP1 homologs clustered according to their phylogenetic subdivision. GLPs from *Linderniaceae*, i.e., CpGLP1, LbGLP1, and LsGLP1, form a separate cluster from GLPs of other eudicots and mosses, monocots, and basal angiosperms. CpGLP1 and the other *Lindernia* GLPs could not be assigned to any of the GLP groups which were previously identified (Fig. S2) (Barman and Banerjee 2015). Amino acid sequence conservation within the three typical structural motifs was very high (Boxes A, B, C; Fig. 1c). However, the N terminus sequence was not conserved (Fig. S1). A KGD motif is found at the N terminus of the germin Box C in CpGLP1 (Fig. 1). This motif is shared with half of the GLP sequences but not present in germins (Barman and Banerjee 2015).

### CpGLP1 is induced by dehydration and ABA

RT-PCR was used to analyze *CpGLP1* transcript levels in untreated *C. plantagineum* plants and plants subjected to dehydration and rehydration. Higher transcript levels were detected in desiccated plants compared to untreated, partially dehydrated, and rehydrated plants suggesting a dehydration-related function for *CpGLP1* (Fig. 2a). The hormone ABA is an important mediator of dehydration-induced gene activation, whereas osmotic stress treatments with high concentrations of the sugar mannitol are often able to mimic the induction of dehydration/desiccation-related genes in *C. plantagineum* (van den Dries et al. 2011). Detached *C. plantagineum* leaves incubated for 2 to 48 h in 100 µM ABA or 0.8 M mannitol showed transient accumulation of *CpGLP1* transcripts (Fig. 2b, c). The level of the *C. plantagineum* elongation factor 1α (*EF1α*) transcripts was used as a control for equal amounts of input cDNA in RT-PCR experiments and did not vary among the group of the samples (Fig. 2). The *CDeT11-24* transcript (Velasco et al. 1998) was amplified in parallel as control for the dehydration and recovery treatments (Fig. 2a).

### CpGLP1 protein accumulates in the cell wall protein fraction of desiccated leaves

The accumulation of the CpGLP1 protein upon desiccation was analyzed with antiserum raised against the recombinant CpGLP1 protein. Several proteins of about 28–29 kDa corresponding to the expected size of CpGLP1 were detected by CpGLP1 antiserum in total protein extracts from untreated, partially dehydrated, desiccated, and rehydrated leaf samples. These bands represent CpGLP1 proteins. Figure S3 demonstrates the specificity of the CpGLP1 antiserum, no protein band was detected with pre-immune serum.

Analysis of the protein sequence with localization tools predicted the presence of a signal peptide at the N terminus suggesting that CpGLP1 is targeted to the apoplast (Fig. 1a). Thus, CpGLP1 protein levels were analyzed in protein fractions enriched for cell wall proteins. Cell wall protein fractions are extracted from cell membranes by sequential extractions with CaCl_2_, EGTA and LiCl solutions (Feiz et al. 2006). Protein blot analysis with protein samples precipitated from CaCl_2_, EGTA and LiCl extracts are shown in Fig. 3. CpGLP1 proteins are abundant in the CaCl_2_ and in the LiCl fractions and accumulate abundantly in the desiccated and rehydrated samples compared to the untreated samples. Control blots with pre-immune serum failed to detect the same protein patterns supporting the specificity of the CpGLP1 antiserum (data not shown).

### CpGLP1 protein interacts with pectin

Because CpGLP1 is localized in the cell wall fraction, it was tested whether CpGLP1 interacts with pectin as a major cell wall component. The interaction of CpGLP1 with pectin was analyzed in ELISA assays using the CpGLP1 recombinant protein and either commercial pectin from citrus or pectin extracted from *C. plantagineum* leaves (Fig. 4). Absorbance detected from wells coated with citrus or *C. plantagineum* pectin was higher than absorbance detected from wells which were not coated with pectin which shows that CpGLP1 binds to pectin (Fig. 4).

### CpGLP1 has SOD activity

SOD activity has been reported for germin-like proteins (Barman and Banerjee 2015). We investigated whether CpGLP1 also has such an enzymatic activity by in-gel activity assays using protein extracts from bacteria overexpressing the CpGLP1 protein. A band corresponding to the size of the recombinant CpGLP1 protein was strongly visible in-gel assays demonstrating that the CpGLP1 has SOD activity (Fig. 5). No corresponding band was detected in the protein sample from bacteria which did not overexpress the recombinant protein. Other weak bands were stained in both overexpressing and non-overexpressing bacteria which are likely to be bacterial SOD proteins.

## Discussion

### CpGLP1 does not belong to previously described GLP subfamilies

GLPs are ubiquitous proteins with members in the angiosperms (monocots, dicots), gymnosperms and mosses. GLPs are predicted to be involved in plant development and stress conditions (Barman and Banerjee 2015; Davidson et al. 2009). During screenings for cell wall proteins putatively involved in dehydration-related mechanisms in *C. plantagineum*, we identify the dehydration-induced GLP gene *CpGLP1* which is coding for a protein that contains the typical sequence structures characteristic for most GLPs (Fig. 1a). Clustering of the putative CpGLP1 homologs from different taxonomical clades and groups indicated a clear separation of CpGLP1 from GLPs belonging to other species outside the Linderniaceae family (Fig. 1b) and from GLPs already assigned to existing groups (Fig. S2). The current GLP classification is based on sequence conservation and was introduced following the analysis of GLPs from different species including major cereals (barley, rice, and maize), soybean, *Arabidopsis*, the moss *P. patens* (Barman and Banerjee 2015; Davidson et al. 2009; Nakata et al. 2004; Zimmermann et al. 2006). According to this classification, GLPs are divided into nine groups which show specific conserved amino acid residues mostly inside the germin Boxes A, B, and C (Barman and Banerjee 2015). With the exception of N-terminal cysteines and the two histidines and the glutamate residues in Boxes B and C, the role of amino acid residues which are often conserved in germin boxes is not known and the relationship between these residues and the protein function is missing. Analysis of the CpGLP1 sequence structures correlates to the phylogenetic analysis thus suggesting a separation of CpGLP1 from previously characterized GLPs. Indeed, the CpGLP1 sequence does not contain all the features from one group but rather shares features from different GLP groups. For example, a phenylalanine (F) is mostly found as the third amino acid in Box A of most GLP groups but this residue is not observed in CpGLP1, GER family 5, 6, GER7 subfamily and some bryophyte subfamily members. Proline and leucine (PL) are found before Box A of CpGLP1 and of GLPs from GER family 3, 4, 7, and 8 but not in GLPs from family 5 and 6. At the fourth position in Box B, there is no asparagine (N) in CpGLP1 as observed for all GLP groups except the GER 2 group. Additionally, a threonine (T) residue at position 8 is not found in CpGLP1 and GLPs from GER 1, and some GLPs from the GER 3 and 4 groups and from the bryophyte subfamily 1 and 2 groups (compare Fig. 1c to Fig. 2 from Barman and Banerjee (2015)). Further investigations are required to understand the link between these sequence variations and the function of CpGLP1 from the different groups.

### CpGLP1 and desiccation tolerance in *C. plantagineum*

Several GLP members are stress modulated and were proposed to participate in plant responses to biotic and abiotic stresses (Davidson et al. 2009; Dunwell et al. 2008). CpGLP1 transcripts and proteins accumulate in dehydrating leaves of *C. plantagineum,* thus they could be linked to the ability of this plant to survive desiccation. Two pathways are involved in the activation of dehydration-related genes in plants, i.e., the abscisic acid (ABA)-dependent and the ABA-independent pathways (Todaka et al. 2015). The plant hormone ABA mediates different stress-related and developmental-related processes in plants (Chen et al. 2020). The transcription of dehydration tolerance genes in *C. plantagineum* can be induced by ABA or mannitol treatments (Bartels et al. 1990; van den Dries et al. 2011). *CpGLP1* transcripts transiently accumulate in response to such treatments (Fig. 2b, c) supporting the involvement of dehydration-related signaling pathways in the regulation of *CpGLP1* transcription/accumulation. ABA-dependent modulation of GLPs has been observed for other stress-induced GLPs suggesting the importance of this hormone for GLP regulation. For example, the expression of *Atriplex lentiformis* GLP (AlGLP) is inhibited by ABA treatments (Tabuchi et al. 2003). All peanut GLPs changed their expression levels (were mostly transiently induced) in response to exogenous applications of ABA in seedlings (Wang et al. 2013). In soybean, GLPs from leaves accumulated in response to ABA and PEG (Li et al. 2016). The expression of the rice germin-like protein OsGLP2-1 is increased in response to ABA and has been shown to be involved in the regulation of seed dormancy (Wang et al. 2020).

### CpGLP1 may participate in the ROS metabolism related to cell wall folding

The regulation of cell wall homeostasis is essential to control cell size and shape during plant development and to protect cell integrity upon stress conditions (Schmidt et al. 2016). Folding of leaves and cell walls is observed in *Craterostigma* spp. upon desiccation. Available data support the hypothesis that this phenomenon is required to reduce the mechanical and oxidative stress generated by the loss of water and thus protect cellular integrity from lethal damage. (Giarola et al. 2016; Jones and McQueen-Mason 2004; Jung et al. 2019; Vicré et al. 1999, 2004). Cell wall folding is accomplished by a combination of constitutive and inducible mechanisms involving the controlled and coordinated rearrangement of molecular and physical linkage of cell wall structures (Jones and McQueen-Mason 2004; Jung et al. 2019; Moore et al. 2008). It has been shown in *C. plantagineum* that these mechanisms may include the specific activation/inactivation of cell wall-modifying enzymes and the accumulation of extracellular Ca^2+^. Indeed, *C. plantagineum* pectin is de-methylesterified during dehydration and this should, together with calcium accumulation, strengthen the cell wall, or lead to pectin cleavage by polygalacturonases and pectate lyases (Jung et al. 2019). Accumulating evidence suggests that extracellular ROS and ROS metabolism could directly and/or indirectly contribute to modify plant cell wall properties and thus could also participate in shrinking mechanisms in *C. plantagineum*. For example, cell wall peroxidases require hydrogen peroxide as co-substrate to mediate cross-linking of cell wall components but can also produce hydroxyl radicals from hydrogen peroxide which are capable of cleaving cell wall polysaccharides (Burr and Fry 2009; Fry 1998; Lindsay and Fry 2007; Passardi et al. 2004). CpGLP1 has SOD activity (Fig. 5) and accumulates in the cell wall of desiccating leaves (Fig. 3b) and thus could participate in the regulation of the ROS metabolism which controls dehydration-related cell wall remodeling. It has been shown that overexpression of the rice germin-like protein 1 in tobacco led to H_2_O_2_ hyper-accumulation and reinforcement of cell wall components (Banerjee et al. 2010). Thus, CpGLP1 could convert stress-induced hydroxyl radicals to H_2_O_2_ which can then act as signaling molecule or serve as a substrate for, e.g., the peroxidase-mediated cross-linking of cell wall components (Marco and Roubelakis-Angelakis 1996). However, the accumulation of hydroxyl radicals in dehydrating *C. plantagineum* leaves and the identification of dehydration-stress induced or active peroxidases remain to be experimentally demonstrated. Bioinformatic analyses of *C. plantagineum* dehydrated and rehydrated transcriptomes pointed to an overrepresentation of peroxidase transcripts in rehydrating leaves (Rodriguez et al. 2010).

We showed that CpGLP1 can weakly, but specifically interact with pectins in vitro and that this interaction is much stronger with pectins extracted from *C. plantagineum* leaves (Fig. 4). Thus, CpGLP1 could possibly interact with specific pectin structures in the cell wall. The interaction with pectin is a novel feature for GLPs and has not been reported for other GLPs so far. CpGLP1 contains a predicted KGD motif. A similar motif, the RGD tripeptide motif is found in animal extracellular adhesion proteins such as fibronectin and vitronectin. The RGD motif of extracellular matrix proteins was shown to function as ligand for integrin receptor proteins (Manjasetty et al. 2005; Ruoslahti 1996). Thus, KGD- and RGD-containing GLPs could interact with other membrane proteins and mediate signal transduction in the extracellular matrix.

A comprehensive study of the apoplastic and membrane-bound proteins involved in the cell wall ROS metabolism is essential to understand how the extracellular ROS homeostasis is controlled in *C. plantagineum* and to establish the role of the ROS and the ROS metabolism in the reversible cell wall folding during desiccation and rehydration.

#### *Author contribution statement*

VG and DB conceived and designed the experiments. SJD identified CpGLP1 in yeast libraries and conducted transcript expression experiments. SM prepared antibodies against CpGLP1. PC performed protein expression analysis and protein–pectin interaction experiments. MK did SOD activity tests. VG conducted bioinformatic analysis and wrote the manuscript. DB supervised the work and corrected the manuscript. All authors read and approved the manuscript.

## Electronic supplementary material

Below is the link to the electronic supplementary material.

Supplementary file1 (DOC 700 kb)
