# Supplementary material for: The dehydration- and ABA-inducible germin-like protein CpGLP1 from Craterostigma plantagineum has SOD activity and may contribute to cell wall integrity during desiccation
Source: Planta. 2020 Oct 12;252(5):84. doi: 10.1007/s00425-020-03485-0 (PMC7550295; doi:10.1007/s00425-020-03485-0)
Supplement: Supplementary file 1 — Supplementary file1 (DOC 700 kb) [file 425_2020_3485_MOESM1_ESM.doc]

**Title**: The dehydration and ABA inducible germin-like protein CpGLP1 from *Craterostigma plantagineum* has SOD activity and may contribute to cell wall integrity during desiccation

Valentino Giarola*, Peilei Chen, Sarah Jane Dulitz, Maurice König, Stefano Manduzio, Dorothea Bartels

Institute of Molecular Physiology and Biotechnology of Plants (IMBIO), University of Bonn, Kirschallee 1, D-53115 Bonn, Germany

*corresponding author

Dr. Valentino Giarola

[valentino.giarola@fmach.it](mailto:valentino.giarola@fmach.it)

+390461615231

ORCID: 0000-0001-6965-4799

present address: Department of Genomics and Biology of Fruit Crops, Research and Innovation Centre, Fondazione Edmund Mach, San Michele all'Adige, Italy

Fig. S1

**
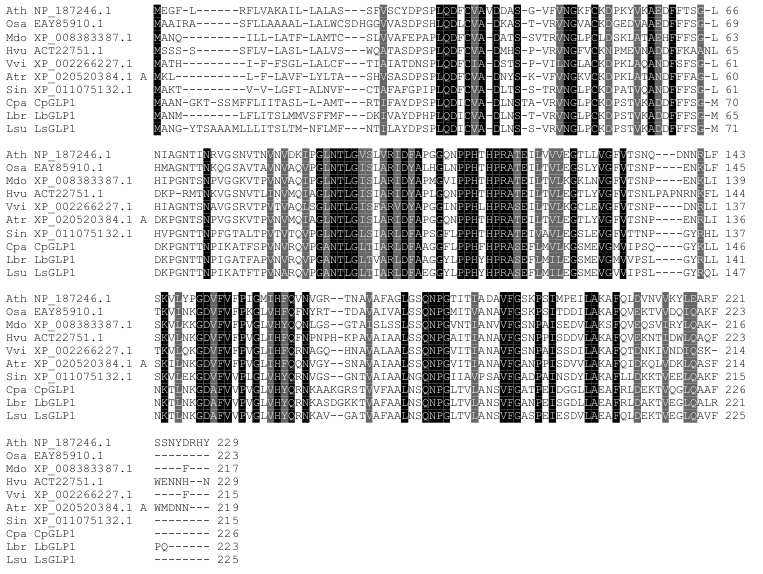
**

Fig. S1 Sequence alignment between CpGLP1 and selected GLPs. Identical (black) and conserved (grey) amino acids are indicated

Fig. S2


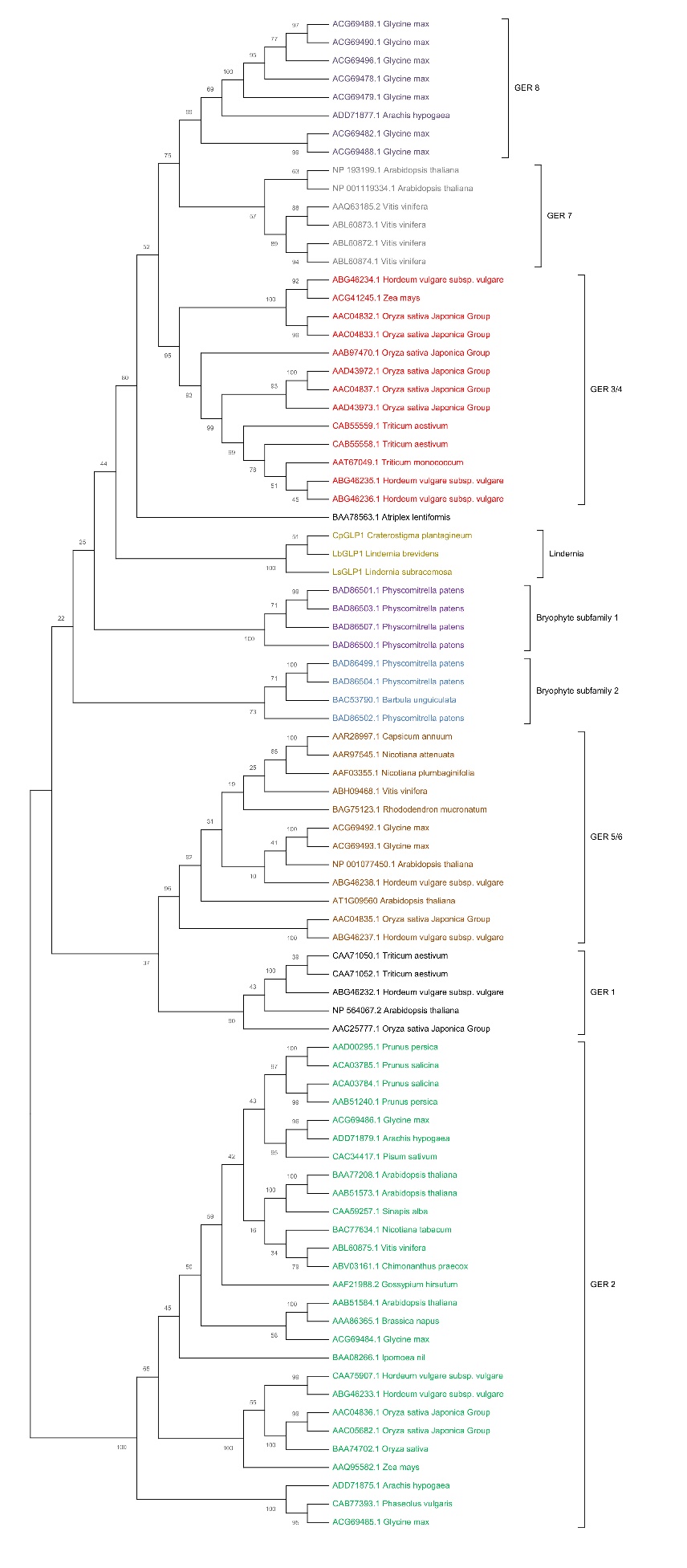


Fig. S2 Phylogenetic analysis of CpGLP1 and GLPs from different groups. GLP sequences previously described in [Barman and Banerjee (2015)](#_ENREF_2) were used for the analysis

Fig. S3

*
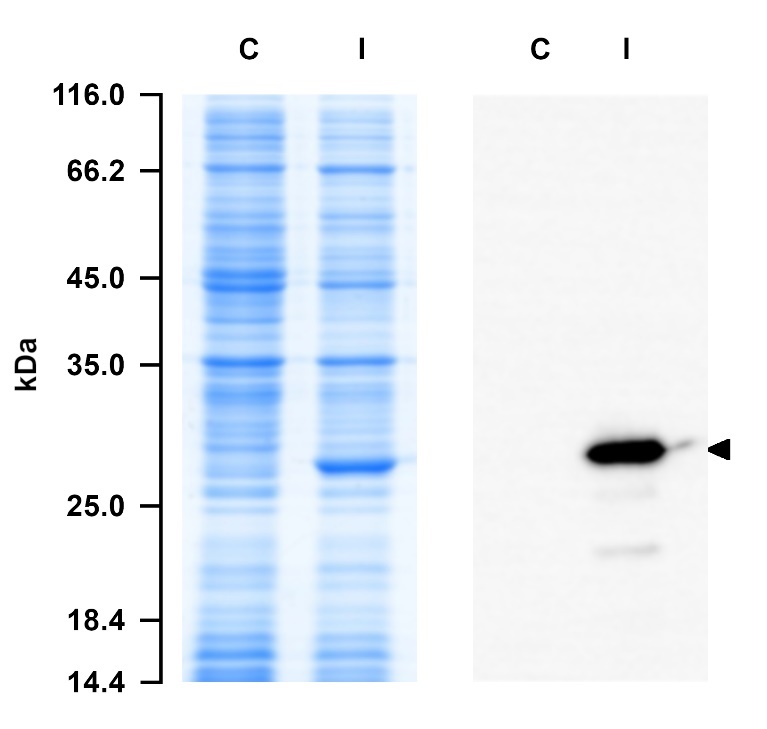
*

Fig. S3 Protein blot analysis showing the specificity of the polyclonal antiserum used to detect CpGLP1. BL21 *E. coli* cells carrying the CpGLP1 (I) overexpressing vector were induced with 1 mM IPTG for 5 hours. Not induced cells carrying the same construct were grown under the same conditions as a control (C). Total proteins were separated by 15 % (w/v) SDS-PAGE. The gel was either stained with Coomassie blue or blotted to a nitrocellulose membrane for protein immunodetection. The CpGLP1 protein is indicated by a black triangle

Fig. S4

GAATTCTTCGCATACGATCCCAGCCCATTGCAAGACATATGCGTAGCAGATCTAAACTCAACTGCAGTAAGAGTCAACGGCTTGCCATGCAAAGATCCTTCGACGGTCAAAGCCGACGACTTCTTCTTCTCAGGCATGGATAAACCTGGGAACACAACTAACCCTATCAAAGCCACCTTCAGCCCAGTCAACGTAAGGCAAGTTCCAGGAGCTAACACGCTGGGGCTCACCATAGCTCGCCTGGACTTCGCCGCAGGGGGGTTTCTCCCGCCGCACTTCCACCCAAGGGCGTCGGAGTTCTTGATGGTTCTAAAAGGTTCCATGGAAGTAGGGATGGTCATACCTAGCCAAGGGTACAAGCTTCTGAACAAAACCCTAAACAAGGGCGATGCTTTCGTCGTCCCCGTCGGCTTGGTTCATTATCAGAGGAACAAGGCGGCGAAGGGGCGGAGCACGGTGGTCTTTGCGGCGCTCAACAGTCAGAATCCCGGACTAACCGTACTTGCCAACAGCGTCTTTGGGGCGACGCCCGAGATCGACGGCGGTTTGCTCGCCGAGGCTTTTCGGTTGGATGAGAAGACTGTTCAAGGGCTTCAAGCTGCCTTTTAATAAAAGTCGAC

Fig. S4 *CpGLP1* sequence used for protein expression in the pET28a(+) vector
